# Tracking Biomarker Responses to Exercise in Hypertension

**DOI:** 10.1007/s11906-023-01252-6

**Published:** 2023-07-10

**Authors:** Eric Trillaud, Philip Klemmer, Steven K. Malin, Uta Erdbrügger

**Affiliations:** 1 Department of Medicine, Division of Nephrology, University of Virginia Health System, Charlottesville, VA USA; 2 Department of Medicine, Division of Nephrology, University of North Carolina, Chapel Hill, NC USA; 3 Department of Kinesiology & Health, Rutgers University, New Brunswick, NJ USA; 4 Division of Endocrinology, Metabolism & Nutrition, Department of Medicine, New Brunswick, NJ USA; 5 The New Jersey Institute for Food, Nutrition and Health, Rutgers University, New Brunswick, NJ USA; 6 Institute of Translational Medicine and Science, Rutgers University, New Brunswick, NJ USA; 7 Footwear R&D, On AG, Zurich, 8005 Switzerland

**Keywords:** Hypertension, Aerobic exercise, Resistance exercise, Oxidative stress, Inflammation, Myokines, Novel biomarkers, Biochemical biomarkers

## Abstract

**Purpose of Review:**

Strong evidence is evolving that physical exercise prevents hypertension and reduces blood pressure in patients with pre- and manifest HTN. Yet, identifying and confirming the effectiveness of exercise are challenging. Herein, we discuss conventional and novel biomarkers such as extracellular vesicles (EVs) which may track responses to HTN before and after exercise.

**Recent Findings:**

Evolving data shows that improved aerobic fitness and vascular function as well as lowered oxidative stress, inflammation, and gluco-lipid toxicity are leading biomarkers considered to promote HTN, but they explain only about a half of the pathophysiology. Novel biomarkers such as EVs or microRNA are providing additional input to understand the complex mechanisms involved in exercise therapy for HTN patients.

**Summary:**

Conventional and novel biomarkers are needed to fully understand the integrative “cross-talk” between tissues to regulate vasculature physiology for blood pressure control. These biomarker studies will lead to more specific disease markers and the development of even more personalized therapy in this field. However, more systematic approaches and randomized controlled trials in larger cohorts are needed to assess exercise effectiveness across the day and with different exercise types.

## Introduction

Arterial hypertension (HTN) is a major modifiable risk factor for cardiovascular diseases (CVD) affecting over a billion people worldwide. Its prevalence will increase by 50% in the next 30 years assuming that current behaviors (e.g., sedentary, excess sodium and caloric intake, smoking, poor sleep) continue unabated [1]. Yet, prevention and treatment of HTN remain challenging. Only 50% of hypertensive patients have controlled HTN. Lifestyle modification consisting of regular physical activity (e.g., 30 min/day) most days of the week is considered a key factor in the prevention and management of HTN. Exercise can also improve long-term survival [2–4]. Despite these well-known effects, only 5–15% of US adults meet physical activity recommendations by the World Health Organization [5]. Tracking response to these interventions with biomarkers might be important to guide for hypertension treatment and even the personalization of care. As HTN is a silent disease, sensitive and early biomarkers are also needed to better describe early hypertensive target end-organ damage.

Biomarkers have already been of great interest in sports for athletes. They have been utilized to measure performance (e.g., lactate, cortisol) and progress and to identify overtraining [6]. During the last decade, growing interest has focused on biomarkers aimed at evaluating health-related factors associated with regular physical activity and sport [7]. These biomarkers of fitness, vascular function, oxidative stress, and inflammation as well as gluco-lipid toxicity have also the potential to reveal possible adverse physiological consequences of exercise. Nevertheless, the exact mechanism(s) by which exercise affects HTN favorably is not totally clear.

Many exercise studies in HTN are observational, self-reported, and, therefore, biased. Tools are still needed to understand minimal and optimal frequency, intensity, and duration of exercise. In addition, the type/modality of exercise required for prevention or treatment of HTN awaits determination. In addition, several effect modifiers must be considered. These include sex, obesity, lifestyle, and pre-existing conditions [8]. Reference standards of these confounders are lacking for different groups, including athletes and populations with known cardiovascular or metabolic diseases.

This article is aimed at briefly summarizing the effects of exercise on the physiology in HTN. In addition, current recommendations for exercise prescription for prevention and treatment of HTN will be outlined. It is important to note that most research to date has focused on aerobic exercise. Future emphasis should include more research on resistance exercise and/or the combination of aerobic and resistance exercise [9, 10]. As such, aerobic exercise will be the focus unless otherwise specified. We will then explore different types of conventional biomarkers already used, as well as provide an overview for the novel biomarkers referred to as extracellular vesicles (EVs) to provide a basic and clinical perspective in this field.

## Physiology of Exercise in HTN

Regional vasodilation in active muscle groups is of prime importance to deliver sufficient oxygen to muscles during exercise. Local control of vascular vasodilation is initiated by the effect of increased cardiac output (CO) on shear stress on arterial endothelium. This signal promotes NO synthesis via endothelial nitric oxide synthetase (eNOS) leading to regional vasodilation. These regional events in the vascular beds of contracting muscles allow blood flow to match metabolic demand. Although CO increases significantly during exercise, blood pressure does not as much [11]. Nevertheless, the adaptations of blood pressure to exercise are complex and different responses to exercise exist in different individuals.

A *pre-exercise response* reflects adaptations of the CO before the beginning of any exercise, called the “mild mental stress” [12]. Compared with rest, heart rate increases usually by a few beats per minute just before the exercise test while the subjects are preparing themselves mentally for exercise. This is explained by a simultaneous vagal withdrawal and release of norepinephrine by the nerve endings. Increase of blood flow is the first adaptive response that helps the redistribution from the circulatory system to the active muscles (responsible for the movement).

An *immediate response* during exercise leads to further increase of blood pressure and redirects the flow to provide an appropriate level of increased blood flow to active muscles [13]. CO increases as does oxygen extraction. Of note, the diastolic blood pressure remains flat or even decreases but the systolic BP continuously increases. This increase, along with regional NO-facilitated vasodilation, increases blood flow to contracting muscles. Also of interest, some patients have a *hypertensive response* to exercise (HRE) defined as the delta between peak and baseline systolic blood pressure (SBP > 60 mmHg for men and > 50 mmHg for women) [14]. This may reflect suboptimal eNOS-induced vasodilation. Excess blood pressure elevation in response to exercise has been linked with increased CVD and mortality [15•]. In contrast, a *post-exercise hypotensive response* occurs in 75% of patients and can last up to 22 h after exercise. This is caused by reduced norepinephrine levels and thus by inhibition of sympathetic activity and reduction in circulating angiotensin II, adenosine, and endothelin levels and their receptors in the central nervous system. These events lead to decreased total peripheral resistance (TPR) and increased baroreflex sensitivity [16]. The post-exercise hypotensive response is dose dependent and stronger in patients with higher baseline blood pressure.

**Fig. 1:**
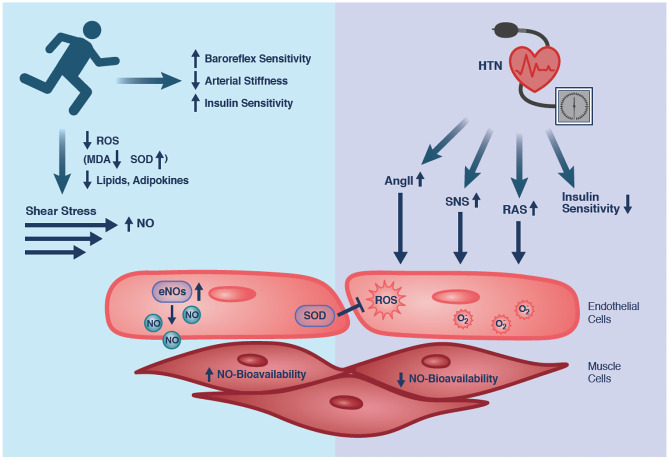
Physiology of exercise on HTN. Long-term and regular aerobic physical exercise improves endothelium-dependent vasorelaxation through an increase in the release of nitric oxide (NO) in normotensive as well as hypertensive subjects [116]. Increased NO bioavailability after exercise may enhance endothelial-dependent vasorelaxation through the endothelial NOS (eNOS) and NO signaling pathway. Aerobic exercise has been shown to be effective in deceasing NO scavenging by ROS [42]. In addition, increased antioxidant activity has been found after exercise reflected in increased levels of superoxide dismutase (SOD) and catalase activities [117]. Exercise also changes vascular tone by reducing local RAS activation and levels of endothelin I and angiotensin 1 receptor. In addition, blood pressure control is determined by the autonomic nervous system. Normally, the sympathetic nervous system is overstimulated in HTN and parasympathetic control decreased. Exercise can reset baroreflex sensitivity in the aortic arch and carotid sinus whose sensitivity is increased in HTN. This effect is likely due to a mechanical or neural component. Thirdly, exercise leads to metabolic and inflammatory changes. Lipid and glucose metabolism improves vascular structure and function. Improvement of insulin sensitivity resets the vascular response to NO. Exercise also normalizes levels of pro-inflammatory cytokines/myokines and endocrine-inflammatory mediators including cortisol and leptin, which can also affect NO bioavailability

A *chronic physiologic response* and thus the preventive or antihypertensive effect of exercise are mediated through four major effects including improvement of endothelial function, vascular structural changes, metabolic/oxidative/inflammatory changes, and modulation of the nervous system stimulation. Different modalities such as aerobic or resistance training or combinations of modalities can be used to achieve these changes. We will refer the reader for in-depth understanding of the different effects of each exercise modality to other review articles [17]. The four major chronic physiological responses with exercise are also visualized in Fig. 1 and described in detail in the legend.

## Prescription of Exercise in HTN

There is significant evidence that an inverse dose response between cardiorespiratory fitness and incidence of HTN is independent of baseline cardiorespiratory fitness [18]. This suggests that any type of exercise at any stage can potentially improve outcome. In addition, regular physical activity is highly effective in improving aerobic fitness and reaches a 30–40% reduction in the risk of heart disease in all populations [19]. It is also established that lowering systolic blood pressure with pharmacological intervention by only 5 mmHg will reduce risk, for example, for stroke by 14% and myocardial infarction by 9%. This degree of blood pressure lowering can easily be achieved with exercise in patients with HTN (8.7 mmHg for endurance, 7.2 mmHg for resistance exercise, and 13.5 mmHg for combined exercise) [20, 21].

WHO: 150 min/week of moderate aerobic or 75 min/week vigorous exercise combined with muscle strengthening 2 days/week.

AHA 2017: “BP-lowering effects have been reported with lower- and higher-intensity exercise and with continuous and interval exercise training. Meta-analyses suggest isometric exercise results in substantial lowering of BP” [22].

ACSM 2018: aerobic exercise 5–7 days/week, plus resistance exercise 2–3 days/week and flexibility exercise 2-3 days/week, ranging from moderate to vigorous (50–70% VO_2_max) [22].

More recently, the European Association of Preventive Cardiology (EAPC) and the ESC Council on HTN provide a consensus document to personalize prescription in the prevention and treatment of arterial HTN [23•]. Biomarkers have the potential to guide this personalization of treatment.

In summary, abundant evidence shows that prescribing moderate exercise can lower blood pressure and even prevent HTN [24]; however, many knowledge gaps exist. It is not known exactly what the minimal and optimal amount of aerobic exercise is to prevent HTN. Furthermore, how different modes of exercise impact HTN remains to be determined with regard to frequency, intensity, and duration of exercise. More recently, interest has grown in efforts to understand how even breaking up sedentary behavior can modify health independent of exercise. This approach is consistent with timing physical activity before or after meals as well as time of day to optimize CVD risk reduction.

## Biomarkers Tracking Response of Exercise in HTN

1. Biomarkers

Biomarkers are objective, quantifiable characteristics of biological processes, measurable accurately and reproducibly. In healthy people, measurement of biomarkers has improved the understanding of the physiology underlying exercise and its potential beneficial effects in disease states. Biomarkers can describe several adaptive processes of the body to exercise in HTN including the effects of acute exercise as well as the impact of long-term training [7]. Most studied biomarkers of exercise response include markers of fitness, vascular function, oxidative stress, and inflammation.

Biomarkers may also be guides of exercise therapy. As such, they have the potential to personalize treatment of HTN. However, it is still unclear if single or multiple biomarkers are needed. The adaptive processes of exercise in HTN are complex and a single biomarker might not be able to capture the broad physiologic dysfunctions associated with HTN. Reference ranges are also not well defined [6]. In this review, we will discuss traditional as well as novel biomarkers of exercise. We will focus on the most studied biomarkers and summarize their important findings in human cohorts. The reader will be referred to other review articles for each biomarker for further in-depth review. Only a limited number of trials exist for novel biomarkers. We will outline the potential of these novel biomarkers.

2. Physical Fitness as a Biomarker

Physical fitness is a primary biomarker to trace the effects of exercise in HTN. In fact, cardiorespiratory fitness (CRF) has been studied as a biomarker to track response of exercise in HTN. CRF is the gold standard to quantify the state of fitness [8]. A recent umbrella review by Pescatello and colleagues included a review of 18 meta-analyses and systematic reviews comprising 594,129 adults [25]. The authors concluded that physical exercise prevents HTN and reduces it in patients with pre- and manifest HTN [25]. CRF was used in many of these studies to assess the fitness of the patients studied. A prospective study with 4.7-year follow-up of 6278 participants confirmed this benefit. Moderate- and high-intensity exercises were associated with 26 and 42% lower risk of HTN [18]. These studies also show that different exercise modalities (aerobic, resistant, combined) are similarly effective to lower blood pressure [20, 25]. This suggests that muscular strength might be another distinct biomarker from that of CRF in contributing to lower HTN and CVD risk. In particular, MacDonald and colleagues demonstrated in a meta-analysis that dynamic resistance training can be a single treatment modality to reduce blood pressure. Noone and colleagues performed the largest meta-analysis to analyze the impact of exercise on blood pressure in hypertensive patients but also compared the antihypertensive effect of exercise to pharmacological treatment of HTN alone [26••]. Overall, 93 randomized controlled trials were assessed. In 32 of those (404 patient with HTN), exercise together with medications lowered effectively blood pressure than controls. They also concluded that antihypertensive medications alone were slightly more effective than exercise; however, there was insufficient evidence to say that first-line antihypertensive medication reduced BP to a greater extent than exercise. In particular, in mild HTN, exercise is therefore still recommended to be the first-line therapy [26••]. For further in depth review, we will refer the reader to several reviews about this topic [8, 18]. It is of note that exercise can also lead to weight loss and thus lower blood pressure through this pathway [27].

3. Biochemical Biomarkers

**Fig. 2:**
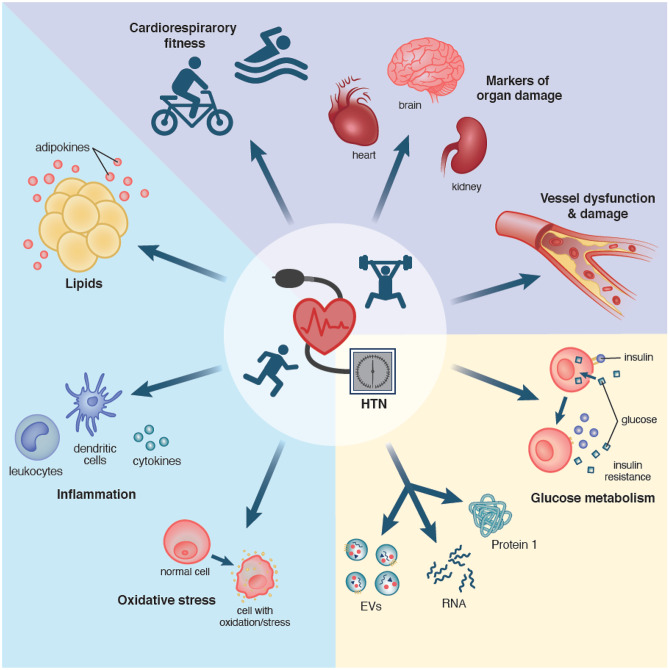
Conventional and novel biomarker categories. This figure summarizes the classes of conventional and novel biomarkers, from classical biochemical biomarkers (blue), markers of exercise fitness, and end-organ damage (purple) to novel biomarkers such as EVs, miRNA, and markers of glucose metabolism (yellow)

Biochemical biomarkers reflecting different physiological pathways such as oxidative stress, inflammation, glucose metabolism, dyslipidemia, and hemostasis have been explored. This is visualized in Fig. 2 as an overview.

*Oxidative stress* is involved in the pathogenesis of HTN and reflects an imbalance between free radicals (reactive oxygen species (ROS), e.g., superoxide, hydrogen peroxide, and hydroxyl radical) and antioxidants (e.g., superoxide dismutase (SOD), catalase (CAT), peroxidases, glutathione, and thioredoxin) [28]. ROS modulate several pathways in HTN leading to decreased bioavailability of NO, increased inflammation, hyperactivity of the sympathetic nervous system, and disturbance of the renin–angiotensin–aldosterone system (RAAS) [29, 30]. Available evidence shows a clear relationship between HTN, oxidative stress/levels of antioxidants, and different types of exercise, also summarized in several recent reviews [31, 32, 33•]. Markers of oxidative stress tested include those of lipid peroxidation, DNA/RNA damage, ROS, antioxidants, and urinary NO metabolites. Therefore, these markers can track responses to exercise. However, each study uses a different panel and detection techniques of biomarkers, thereby making direct comparison across studies difficult. In order to establish them as clinical markers for personalized treatment, reference standards for each biomarker and type of exercise intervention need to be developed.

The effect of different exercise modalities has been tested on markers of oxidative stress. These modalities include aerobic, isometric, and alternative exercise such as walking/Tai Chi/yoga. For example, aerobic exercise for 3, 6, or 12 months has shown to improve blood pressure and levels of NO and superoxide (O_2_) and increase levels of antioxidants [34, 35]. Resistance exercise (isometric or static contractions) on the other hand has been less studied in regard to these biomarkers [36, 37]. In a group of 40 HTN patients, isometric exercise training for 6 weeks decreased ROS and increased antioxidants [37]. Combined exercise training (of aerobic and resistance exercise) and its effect on oxidative stress were also investigated. In a recent single-blinded, randomized control trial of 54 older men and women, combined exercise training showed a better hypotensive and antioxidant effect, which could also be due to a greater adherence and attendance [38].

Some alternative training modes such as walking/Tai Chi/yoga offer more gentle, slow, deliberate movements. They are associated with less lowering of diastolic blood pressure. However, in 140 hypertensive patients assessed with pedometers for 5 consecutive days, those with > 10,000 steps a day had significantly less oxidative stress (higher CAT and SOD levels and low malondialdehyde levels) [39]. A recent meta-analysis of the efficacy of Tai Chi on HTN was conducted in 9 randomized controlled trials with exercise interventions of 1.5 to 6 months. The meta-analysis showed that compared to controls, exercise lowered more effectively systolic and diastolic blood pressure and contributed to higher levels of NO and lower levels of endothelin-1 [40]. However, it is not clear which marker of oxidative stress describes best response to exercise.

Of note, human studies (and also most animal studies) on oxidative stress have only been associative [32, 41–43]. More mechanistic studies are needed. There is also evidence that acute exercise can induce oxidative stress as a result of the inefficiency of the mitochondrial respiratory chain and the increase in fluid shear stress on the endothelium [33•, 44]. Nevertheless, human and animal studies have shown that repeated exposure to mild oxidative stress with exercise may initiate adaptive processes and reduce oxidative stress long-term-wise. Therefore, their levels are not harmful but beneficial with chronic exercise [45]. However, caution is needed for patients who perform extreme sport or have significant CVD and an exaggerated blood pressure response to HTN. This topic is beyond this review and we will refer to other manuscripts [15•].

Persistent *inflammation* in HTN can lead to vascular remodeling and end-organ damage of the vessels, kidneys, heart, and brain [46]. Evolving evidence demonstrates that exercise is anti-inflammatory through different actions, by (a) increasing stress hormones affecting leukocyte trafficking; (b) reducing visceral fat, which in turn decreases release of adipokines; (c) increase in anti-inflammatory myokines (IL-6,-7,-8,-10,-15, irisin, VEGF, and more); and (d) lowering toll-like receptors (TLRs) in immune cells [47]. For example, acute bouts of exercise redistribute lymphocytes to peripheral tissues [48] and activate the innate immune system (natural killer cells, macrophages, neutrophils) [47]. More recently, a study looked at subtypes of WBC s in 31 patients with “pre-HTN” (SBP 130–139) and could only identify CD11cCD16 + monocytes to be elevated, with no change in T-cell levels [49]. However, this group studied only a small study group with pre-HTN or mild HTN. Regarding cytokines, IL-6 stands out as an inflammatory marker as it has a dual function, being pro-inflammatory in sedentary people but anti-inflammatory in more active people. A likely reason for this discrepancy relates to release of IL-6 from adipose versus skeletal muscle in the former and later groups based on physical activity levels [50]. IL-6 is also one of the most studied inflammatory biomarkers in exercise and HTN. Interestingly, pro-inflammatory cytokines can decrease NO bioavailability by stimulation of ROS [51], linking oxidative stress with the immune response.

Two more recent studies randomized HTN patients into groups performing either aerobic exercise or being sedentary. In 90 patients with mild HTN, blood pressure and inflammatory markers (TNFa, IL6) decreased after 3 months of treadmill training. IL-6 levels positively correlated with systolic blood pressure [52]; this finding is also confirmed by Wagner and colleagues [53]. Other investigators studied C-reactive protein (CRP) as an inflammatory marker in 245 male patients with mild to moderate HTN. Eight [8] weeks of interval training decreased CRP and white blood count (WBC) levels. Blood pressure levels correlated also positively with changes in CRP whereas VO_2_max correlated negatively with WBC counts [54]. Overall studies are very heterogeneous using different modes of exercise and study size, different panels of inflammatory biomarkers, and patients with different severity of HTN. Often, these studies do not account for other lifestyle factors such as diet, smoking, and medication effects.

It is also unclear if a panel of inflammatory markers (e.g., cytokines and activated proteins such as CRP) or individual markers provide a powerful biomarker panel to assess the inflammatory response to exercise.

*Myokines and adipokines* belong to a group of circulating proteins/cytokines that communicate with cells in an autocrine and paracrine fashion with cross-talk to other tissues. Skeletal muscle tissue–derived proteins function as myokines (e.g., IL-15 or myonectin) and are involved in energy metabolism, angiogenesis, and myogenesis. However, some myokines are also secreted by adipocytes called adipo-myokines like IL-6 or myostatin. These myokines and in particular IL-6 can counter the harmful effects of pro-inflammatory adipokines (see above paragraph). Yet, there are also “pure” adipokines. For in-depth review of these protein messengers,we will refer the reader to other reviews [55]. These proteins secreted during exercise are also called exerkines and have been tested as biomarkers in HTN. For example, adiponectin levels were tested in 24 overweight patients with grade 1 HTN who underwent 8 weeks of moderate-intensity aerobic exercise and compared to 24 age- and sex-matched patients. Exercise led to weight loss and increase in adiponectin plasma levels. These changes preceded blood pressure changes and therefore might have a predictive function as a biomarker [56]. A more recent study of 52 patients with metabolic syndrome and high-normal blood pressure showed that yoga training with 3 1-h weekly sessions decreased pro-inflammatory adipokines and increased anti-inflammatory ones [57]. Consistent with this work, we showed that interval and continuous exercise training for 2 weeks in older adults with prediabetes who also have high-normal blood pressure lowered mean arterial pressure comparably in parallel with improved total adiponectin and lowered leptin [58]. However, larger studies are needed to confirm these findings.

*Lipoproteins* have been studied as a significant risk factor for CVD. A prospective analysis demonstrated that combining exercise with statin therapy leads to decreased mortality and improved fitness after 10 years of treatment [59]. In addition, a large meta-analysis of 25 randomized controlled studies comparing exercise alone (without medication) to medical therapy with statins showed that high-density lipoprotein (HDL) could be increased with exercise alone [60•]. Interestingly, HDL seems to respond better to exercise than low-density lipoprotein (LDL) and triglycerides (TG); however, most studies by our group and others show that the lipid profile can favorably be improved with exercise in people with normal to elevated blood pressure [61–64], particularly in a dose-dependent manner [65]. Taken together, though the data for LDL and TG are less consistent following exercise, HDL might be the preferred biomarker to assess the effect of exercise.

Different exercise types have been studied on the lipid profile in relation to HTN. A recent randomized controlled trial compared the impact of aerobic vs resistance vs combined aerobic resistance training matched on exercise time (i.e., 60 min/d) in 69 adults (58 + 7 years) with HTN, obesity, and sedentary lifestyle. Combined training showed a greater reduction of a composite score of CVD including lipids [66]. Another study confirmed that combined strength and endurance training can improve HDL levels (not LDL) [67]. For further in-depth review of the effect of exercise on lipids, we will refer the reader to the review by Wang and Xu [68].

*Glucose metabolism* disturbances are documented in people with HTN, and nearly 80% of people with type 2 diabetes have HTN [69]. This highlights a common soil in the pathophysiology. Aerobic exercise training in adults with type 2 diabetes is effective at improving glycemia with fewer daily hyperglycemic excursions and 0.5–0.7% reductions in hemoglobin A1C [70–73]. Furthermore, the US Diabetes Prevention Program (DPP) trial utilized an intensive lifestyle approach with a goal of 5–7% weight loss. Although results showed that T2D risk was reduced by 16% for each 1 kg of body weight loss [74], individuals meeting the PA goal with no weight loss had a 44% reduction in diabetes incidence. These findings support aerobic exercise as an effective tool to manage blood glucose. It should be noted that resistance training may increase lean skeletal muscle mass and reduce A1C threefold more in older adults with T2D compared to a calorie-restricted, non-exercising group that lost skeletal muscle mass [75]. This highlights that either aerobic or resistance exercise is effective at lowering glucose. While debates exist as to whether intensity of exercise induces greater gains in glycemia [76], most studies show that when calories are matched, there are no intensity differences [76]. Recent work has shown, however, that exercise timing may matter for optimal exercise-induced glycemic benefit. In fact, exercise in the post-prandial state and/or in the afternoon may promote better glycemia than pre-prandial or morning exercise [76]. However, it should be noted that pre-prandial and morning exercises still induce benefit on glucose tolerance.

*Hemostatic and fibrinolytic properties* change after acute strenuous exercise and can lead to an increased thrombotic tendency and increased cardiovascular risk in HTN patients [77]. Of note, inflammation is additionally contributing to activation of the coagulation system. This thrombotic tendency occurs in normotensive and hypertensive patients [78], but can be more prolonged and exaggerated in the latter group after exercise. Elevated levels of fibrinogen, increased plasma viscosity, and abnormal clotting activity have been described [77]. However, this increased pro-coagulable state can be improved with HTN treatment with angiotensin receptor blockers [79] and angiotensin-converting enzyme inhibitors [80]. As moderate exercise can lead to platelet activation and aggregation, a recovery period and a gradual progression of exercise intensity are suggested for people at risk for CVD [81, 81]. Fortunately, long-term exercise training will likely lead to sustained benefits regarding the fibrinolytic activity. Nevertheless, it is unclear which intensity and duration of exercise seem to lower the thrombogenic activity (TA) best in HTN and other CVDs. To our knowledge, a specific hemostatic or fibrinolytic marker has not been identified to assess an individual patient’s risk for cardiovascular complications due to thrombotic tendency [77]. For a very detailed analysis on effects of exercise on the coagulation system in normotensive and hypertensive patients, we will refer the reader to a recent review by Braschi [77].

### Other Non-traditional Biomarkers

*Hypertensive target end-organ damage* should ideally be detected in its early stages. As HTN is a silent disease, early biomarkers are crucially needed, but unfortunately are lacking. Screening for left ventricular hypertrophy (cardiac damage), HTN retinopathy (eye damage), or elevated albuminuria or increased serum creatinine levels (kidney damage) is routinely performed; however, when detected, it indicates that significant end-organ damage is already present. However, a recent meta-analysis of 13 randomized controlled studies showed that exercise therapy could benefit non-dialysis CKD patients by increasing estimated glomerular filtration rate (eGFR) while reducing systolic and diastolic blood pressure and body mass index [82]. Improvements of other end-organ damage such as cardiac fibrosis and cardiac remodeling in HTN patients after exercise have also been studied [83, 84].

*Endothelial function* (flow-mediated dilation, FMD) and *arterial stiffness* (augmentation index AI and pulse wave velocity PWV) are mostly measured by experienced hands in clinical research labs, but provide pre-clinical insight towards vascular damage. A meta-analysis by Ashor and colleagues showed that exercise improves endothelial function (measured by FMD) and arterial stiffness (measured by AI, PWV) with a dose response between exercise intensity and improvement of vascular changes [85••]. Interestingly, while this was also confirmed in patients with pre-HTN and HTN [86], not all studies agree. Indeed, we have shown that interval and continuous aerobic exercise induce similar improvements in AI during the post-prandial state in older adults with obesity and prediabetes [87]. Moreover, we have observed no effect of either a single bout [61] or 2 weeks of exercise [88] by intensity in adults with prediabetes when using an oral glucose tolerance test. In contrast, a recent work we performed in middle-aged adults with obesity showcases that a single bout of exercise at 65% of VO_2_max can improve large conduit artery diameter and microcirculatory blood flow in response to insulin during euglycemic conditions [89]. Given that shear stress is considered a key stimulus for NO, these later results suggest that exercise may influence how endothelial cells respond to insulin prior to shear stress. Indeed, we have seen that insulin acts directly on central hemodynamic and AI in adults with metabolic syndrome who have HTN on or off medications [90]. However, these “biomarkers” of vascular dysfunction and damage are not yet routinely executed in clinical practice and most works to date have focused on fasting measures only in the literature. Also, few studies have assessed microvascular function compared with large conduit arteries. This is another important consideration as large conduit artery function may clinically relate better to atherosclerosis whereas microvascular function connects with end-organ damage [91]. Additional work characterizing the “fed” state is needed since post-prandial glucose and lipids are known to induce CVD risk to a greater extent than fasting milieus [92–94].

### Novel Biomarkers

*Extracellular vesicles* are evolving as novel cell to cell and organ to organ communicators in exercise physiology [95•, 96]. They are also tested as novel biomarkers in HTN [96, 97]. These submicron vesicles can either derive from the vesicular membrane of cells through a blebbing process or are released from multivesicular bodies from within the cell into the extracellular space [98]. They are found in all types of bodily fluids, but also in the interstitial space, e.g., of skeletal muscles [99]. Exercise provides a strong stimulus for EV release; however, their phenotype and cargo depend on exercise mode and time of investigation [96]. Of note, the skeletal muscle, the largest organ in the body, is also seen as an endocrine organ, releasing myokines and exerkines. EVs are likely delivery molecules of these exerkines and might play an important role in mechanism and adaptation to exercise as messengers have local and systemic effect [100]. Skeletal muscle–derived EVs have been found to increase in particular after exercise [101, 99]. EVs, named also ExerVs, have also been found to be involved in exercise adaptations in angiogenesis, immune signaling, glycolysis, and transportation of myokines [96, 102•]. Acute and chronic effects of exercise and its effects on EVs have been studied only in a few studies in HTN. For example, we showed in individuals with mild HTN and very poor fitness (VO_2_peak = about 15 ml/kg/min) that EVs were higher compared with people who were considered to have similar blood pressure but poor fitness (VO_2_peak = about 25 ml/kg/min) [103]. These results may have clinical relevance since EVs correlated with AI (a surrogate for arterial stiffness/pulse waveforms) and 2-h glucose levels as well as low HDL. Interestingly, we followed up this work by examining the effect of exercise intensity for 2 weeks on EVs in older adults with obesity and prediabetes who had high-normal blood pressure. The results showed prior to clinically meaningful weight loss that interval exercise lowered endothelial-derived EVs (CD105) compared with continuous exercise matched on energy expenditure [87]. Our work is consistent with Kim et al. who studied adults with pre-hypertension and found that the 3 days per week of 40 min of exercise at 65% predicted heart rate for 6 months decreased endothelial-derived EV counts (CD31+/CD42a- and CD62E+) [104]. Babbitt and colleagues confirmed this finding by showing that endothelial-derived EVs and markers of inflammation also decreased after 6 months of aerobic exercise training in African Americans with increased risk for HTN [105]. These studies together though likely included only larger EVs as conventional flow cytometry was used to characterize EVs. Larger studies and more comprehensive EV characterization to include also small EVs are needed to optimize treatment towards the exact pathophysiology of HTN [97, 106].

*MicroRNAs* have been studied with increased interest in exercise training of HTN patients [107]. MicroRNAs (miRNAs) are a class of non-coding RNAs that play important roles in post-transcriptional regulation of gene expression. Many different miRNAs are dysregulated in HTN and are associated with pathophysiological mechanisms involved in HTN including vascular dysfunction and activation of the renin–angiotensin–aldosterone system or autonomic nervous system [107]. MiRNA profiles are dynamic and different in the acute and chronic phase of exercise. Several studies have looked at how exercise improves HTN through specific miRNAs in the heart, vascular system, and skeletal muscle, all players of HTN pathophysiology [118]. Interestingly, different miRNAs have opposite expression profiles in HTN and after exercise, indicating their possible regulatory role. For example, microRNA-29b, which regulates VEDG and collagen genes, was highly expressed in HTN, but reduced with exercise [108]. Mir-324, however, was downregulated in HTN, but increased after aerobic exercise. MiR 324 regulates mitochondrial function [109]. Studies of other small RNA and epigenetics in ET and HTN have not been reported. Many of these findings need to be validated and also different modalities of exercise studied.

*Proteomics* has also been utilized to study the different molecules providing tissue cross-talk during exercise. Early studies linked changes in function with changes in protein expression and post-translational modification which also showed the potential to uncover novel mechanisms underlying benefits of physical activity [110]. More recently, the interest focused to study the EV proteome in exercise [102•]. Whitham and colleagues studied the EV proteome in healthy humans following a 1-h bout of cycling exercise. They observed an increase of over 300 proteins in the circulation and identified novel candidate myokines released during exercise [102•]. To our knowledge, studies looking at the proteome of EVs after exercise in hypertensive humans have not been studied; however, it would allow deeper analysis of EV protein cargo and identification of additional biomarker candidates.

## Outlook/Conclusion/Summary

Strong evidence is evolving that physical exercise prevents HTN and reduces blood pressure in patients with pre- and manifest HTN [25]. Yet, the minimal and optimal frequency, intensity, and/or duration as well as type of exercise in HTN remain to be elucidated. Further, how exercise interacts with diet, sleep, and/or medication remains understudied. Examining additional behaviors surrounding exercise is critical towards understanding how to maximize the effects of exercise to lower blood pressure. This said, identifying and confirming effectiveness of exercise are challenging. This review has discussed conventional and novel candidate biomarkers that can reflect the beneficial effect of exercise on HTN. While other evolving fields include the study of proteostasis, autophagy, and metabolomics, these studies are also in their infancies. Collectively, biomarkers reflect not only the complex mechanisms involved in exercise therapy for HTN patients but also the integrative “cross-talk” between tissues to regulate vasculature physiology for blood pressure control. More systematic approaches and randomized controlled trials in larger cohorts are needed to assess exercise effectiveness across the day. Indeed, much of the research on blood pressure exists from clinical readings (e.g., single morning read). More work is needed to understanding blood pressure across the 24-h period given that nocturnal blood pressure is an independent risk factor from that of clinical blood pressure for CVD [111]. An important consideration for the field in understanding mechanism(s) of exercise on HTN will be to consider novel biomarkers since classic biomarkers (e.g., glucose, lipids) may only predict about 20–40% of CVD risk [112–115]. As such, to implement more cost-effective strategies to combat CVD, we propose that novel biomarkers such as EVs or microRNA will lead to more specific disease markers and the development of personalized therapy in this field.
